# Anticoagulant Protective Effects of Sulfated Chitosan Derived From the Internal Bone of Spineless Cuttlefish (Sepiella inermis)

**DOI:** 10.7759/cureus.64558

**Published:** 2024-07-15

**Authors:** Megha Poolakkal Sajith, Annathai Pitchai, Pasiyappazham Ramasamy

**Affiliations:** 1 Physiology, Saveetha Dental College and Hospitals, Saveetha Institute of Medical and Technical Sciences, Saveetha University, Chennai, IND; 2 Prosthodontics and Implantology, Saveetha Dental College and Hospitals, Saveetha Institute of Medical and Technical Sciences, Saveetha University, Chennai, IND

**Keywords:** thrombin, sulfated chitosan, innovation, chitosan, anticoagulant activity, internal bone

## Abstract

Background

This study investigated the anticoagulant properties of sulfated chitosan derived from the internal bone of the spineless cuttlefish *Sepiella inermis*. Chitosan, a biopolymer, is used in various biomedical applications including anticoagulation. Sulfation of chitosan enhances its biological activity, making it a potential therapeutic agent. This study explored the efficacy of sulfated chitosan in preventing blood clot formation to provide a novel anticoagulant alternative.

Objectives

This study aimed to synthesize and characterize the anticoagulant properties of sulfated chitosan extracted from the internal bone of the spineless cuttlefish *S. inermis* using Fourier Transform Infrared Spectroscopy (FTIR), Field Emission Scanning Electron Microscopy (FESEM), and X-Ray Diffraction (XRD) and evaluate the anticoagulant properties of sulfated chitosan extracted from the internal bone of spineless cuttlefish *S. inermis*.

Materials and methods

Chitin and chitosan were extracted from the cuttlebone of a specimen of *S. inermis*, and sulfated chitosan was synthesized by sulfation of chitosan. Sulfated chitosan was subsequently used to evaluate its anticoagulant properties using tests such as activated partial thromboplastin time (APTT) and prothrombin time (PT). Characteristic investigations were conducted, including FTIR, FESEM, and XRD analyses.

Results

The results of this study suggested the possibility of using *S. inermis *internal bone as an unconventional source of natural anticoagulant that can be combined with biomedical applications. Anticoagulant activity measured using APTT and PT showed that sulfated chitosan was a strong anticoagulant.

Conclusion

We examined the anticoagulant activity of *S. inermis* extract using thrombin and activated partial thromboplastin times. Our results demonstrated the heparin-like anticoagulant action of the extracted sulfated chitosan, suggesting that it may be a great alternative anticoagulant treatment.

## Introduction

Chitosan is characterized by biocompatibility, biodegradability, and nontoxicity. Owing to these characteristics, chitosan has been the subject of intensive studies to improve its functional qualities. Adding sulfate groups to chitosan to form chitosan sulfate is one such modification. This alteration provides chitosan with bioactive capabilities (anticoagulant and antioxidant) and changes its physicochemical properties, making it a promising candidate for a range of biological applications (adsorbing metal ions, drug delivery systems, blood compatibility, and antimicrobial activity) [1,2]. Marine organisms are an important, but not fully used, source of sulfated polysaccharides and have special chemical structures and properties. These marine polysaccharides can cause illness but are safer than those found in land animals [3,4]. Polyanionic polymers, known as sulfated polysaccharides, are found in nature. Where polysaccharides originate and how they are removed can change how they are made and what they can do in the body. They have many different effects on the body, such as preventing blood clotting, reducing inflammation, protecting cells from damage, improving the immune system, and fighting cancer. This is because they can interact with the cells and proteins in the body. This means that they can be used in many medical and drug applications [5].

The use of chitosan is restricted by its remarkably low solubility, which is caused by its highly crystalline structure promoted by hydrogen bonds and acetamido groups. Thus, to produce high-quality derivatives, the structure of chitosan must be altered. Chitosan has been successfully used to modify sulfated chitosan and produce novel biofunctional materials chemically. Because the structure of sulfated chitosan is similar to that of heparin, it clearly exhibits anticoagulant properties [6]. Sulfated chitosan, which has strong anticoagulant properties, has been isolated from marine organisms in several studies. The goal of these investigations was to identify sulfated chitosan belonging to the glycosaminoglycan (GAGs) family as a substitute for heparin as an anticoagulant [7,8]. Heparin substitutes are preferred for the following reasons. For instance, because heparin is derived from the intestines of pigs and the lungs of cows, several religious communities are against its use. Heparin is also associated with several fatal illnesses. According to a recent study by Liu et al., heparin-induced thrombocytopenia poses a significant risk of death in critically ill COVID-19 patients receiving this medication [9,10]. *Sepiella inermis*, a spineless cuttlefish, lives in the shallow Indo-Pacific coastal waters. The capacity of this species to quickly change color and texture helps it disguise and communicate. Asian countries gather *S. inermis* for its flesh and ink, thus making it financially valuable.

Abnormal build-up of blood clots in the blood arteries or thrombosis is a significant medical condition that can result in heart attacks, strokes, and pulmonary emboli. The serine protease enzyme thrombin is a major modulator of thrombus formation and is essential for the blood coagulation cascade. One potential strategy for managing and preventing thrombotic diseases is inhibition of thrombin activity. Conventional anticoagulant treatments have several drawbacks, including limited therapeutic windows and bleeding concerns [11]. Thus, a key field of study is to investigate alternative anticoagulants that efficiently target thrombin while avoiding side effects. Owing to the special qualities arising from the addition of sulfate groups, chitosan sulfate has demonstrated promise as an anticoagulant. Research has indicated that chitosan sulfate strongly interacts with thrombin, which inhibits it. The positively charged areas on the surface of thrombin and the negatively charged sulfate groups on chitosan sulfate were assumed to interact electrostatically to cause this interaction [12]. Chitosan derivatives have biological potential, but little is known about the production and anticoagulant properties of sulfated chitosan from *S. inermis* internal bones. This source also lacks structural characterization and comparative in vitro anticoagulant tests using the APTT and PT kits. Hence, the present investigation aimed to synthesize sulfated chitosan from the internal bone of *S. inermis*, structurally characterized it using Fourier Transform Infrared Spectroscopy (FTIR), Field Emission Scanning Electron Microscopy (FESEM), and X-Ray Diffraction (XRD), and evaluated its in vitro anticoagulant potential using activated partial thromboplastin time (APTT) and prothrombin time (PT) kits.

## Materials and methods

Extraction of chitin and chitosan

Through demineralization and deproteinization, chitin was extracted from the internal bone of *S. inermis*. Thus, chitin from internal bones was transformed into chitosan using deacetylation, which involves 40% aqueous NaOH [13].

Preparation of sulfated chitosan

The Xing technique [14] was used for the sulfation of chitosan. To obtain gelatinous chitosan, 50 ml of a chitosan solution in a dimethylformamide (DMF) combination was placed in a 500 ml flask with three-necked bottoms and shaken. After running the reaction for 1-2 h at a suitable temperature of 40-60 °C, 300 ml of 95% ethanol was added to the precipitate. The solution was filtered using a Buchner funnel at low pressure. After cleaning the precipitate with ethanol, it was dissolved in deionized water. NaOH (2 M) was used to reduce the pH to 7-8. Using a 12,000 Da Molecular weight cutoff dialysis membrane, the solution was dialyzed against distilled water for 48 h. Subsequently, the dialyzed product was lyophilized and concentrated.

Characteristic analysis of sulfated chitosan

The sulfated chitosan was subjected to standard analyses, including Fourier Transform Infrared Spectroscopy (FTIR), Field Emission Scanning Electron Microscopy (FESEM), and X-ray diffraction analysis.

FTIR analysis for sulfated chitosan

The FT-IR spectra of powdered sulfated chitosan samples taken from *S. inermis* internal bone were measured using a Bruker ALPHA II (Berlin, Germany) ​​​​​​FT-IR spectrometer.

FESEM analysis for sulfated chitosan

The morphological characteristics of sulfate chitosan were investigated using (field-emission scanning electron microscopy (FESEM; JEOL model 6390,** **Tokyo, Japan). Electron scattering was focused on a tiny probe for FESEM, which was then raster-scanned or methodically moved across a relatively small rectangular area. The interaction between the beam and sample produces a variety of observable signals, including internal currents, secondary electrons, and photon emission.

XRD for sulfated chitosan

An X-ray diffractometer (XRD-6000, Shimadzu XRD Diffractometer, Shimadzu Corporation, Chiyoda-ku, Tokyo, Japan) was used to study the structural characteristics of sulfated chitosan. The diffraction angle, 2θ, and orientation of the specimens were calculated to estimate their X-ray diffraction intensity. This diffraction pattern can be used to determine the structural properties of the material as well as the size and direction of the crystallites, which are tiny pieces of the crystalline structure.

*In vitro* anticoagulant activity

Activated partial thromboplastin time and prothrombin time are two tests used to determine how well-sulfated chitosan works as a blood thinner. For one minute, 100 μL plasma containing sulfated chitosan and heparin sulfate was maintained at 37 °C. After adding 100 μL cow blood, the mixture was incubated at 37 °C. After waiting for three minutes, the mixture was mixed with 100 μL of a warmed solution containing 0. 25 The time taken for the blood to clot was measured and compared with the usual time taken for blood to clot. The clotting activity was measured in units (IU). The thromboplastin reagent was mixed with plasma containing different concentrations of sulfated chitosan after incubation for five minutes. Simultaneously, the time taken for blood to clot was recorded [15].

Statistical analysis

Variables are expressed as mean ± SD. Analysis of variance (P < 0.05) was used to evaluate the data, and Duncan's multiple range test was used to separate means. SPSS 25 (IBM Corp., Armonk, USA) and Microsoft Excel (Microsoft Corporation, Redmond, USA) were used to analyze the results.

## Results

FTIR analysis

**Figure 1:**
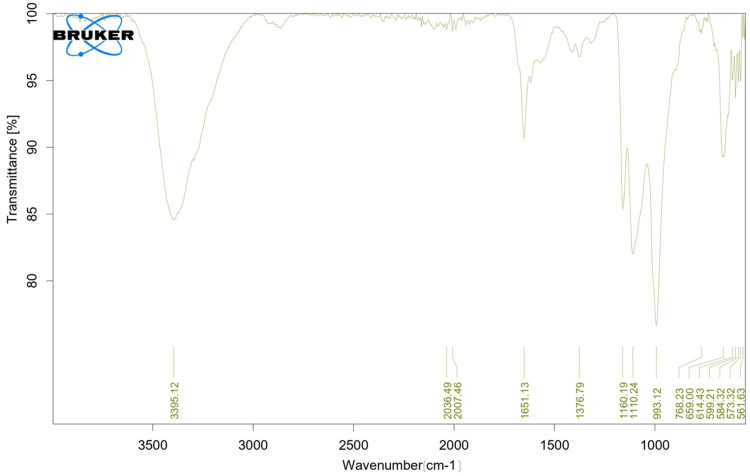
Fourier transform-infrared spectral analysis of sulfated chitosan from internal bone of Sepiella inermis

Because of the hydroxyl stretching vibration of sulfated chitosan, a large intensity band at 3395.12 cm^−1^ was predicted in the infrared spectrum (Figure 1). The stretching vibrations of the CHO and C=O bonds were attributed to absorptions at 1651.13 cm^−1^. The FTIR spectrum showed significant absorption bands in the 1160.19 cm^−1^ region, indicating asymmetric stretching of the N-S=O bond.

FESEM analysis

**Figure 2:**
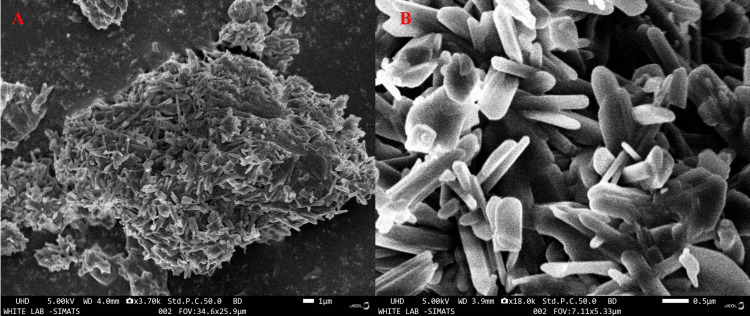
Field Emission Scanning Electron Microscopy image of sulphated chitosan from internal bone of Sepiella inermis (A,B)

The sulfate chitosan is shown in the FESEM images to have a smooth, nonporous fibril structure (Figure 2). The rod-like form of sulfate chitosan, which has several potential biological uses, was further verified by FESEM.

XRD analysis

**Figure 3:**
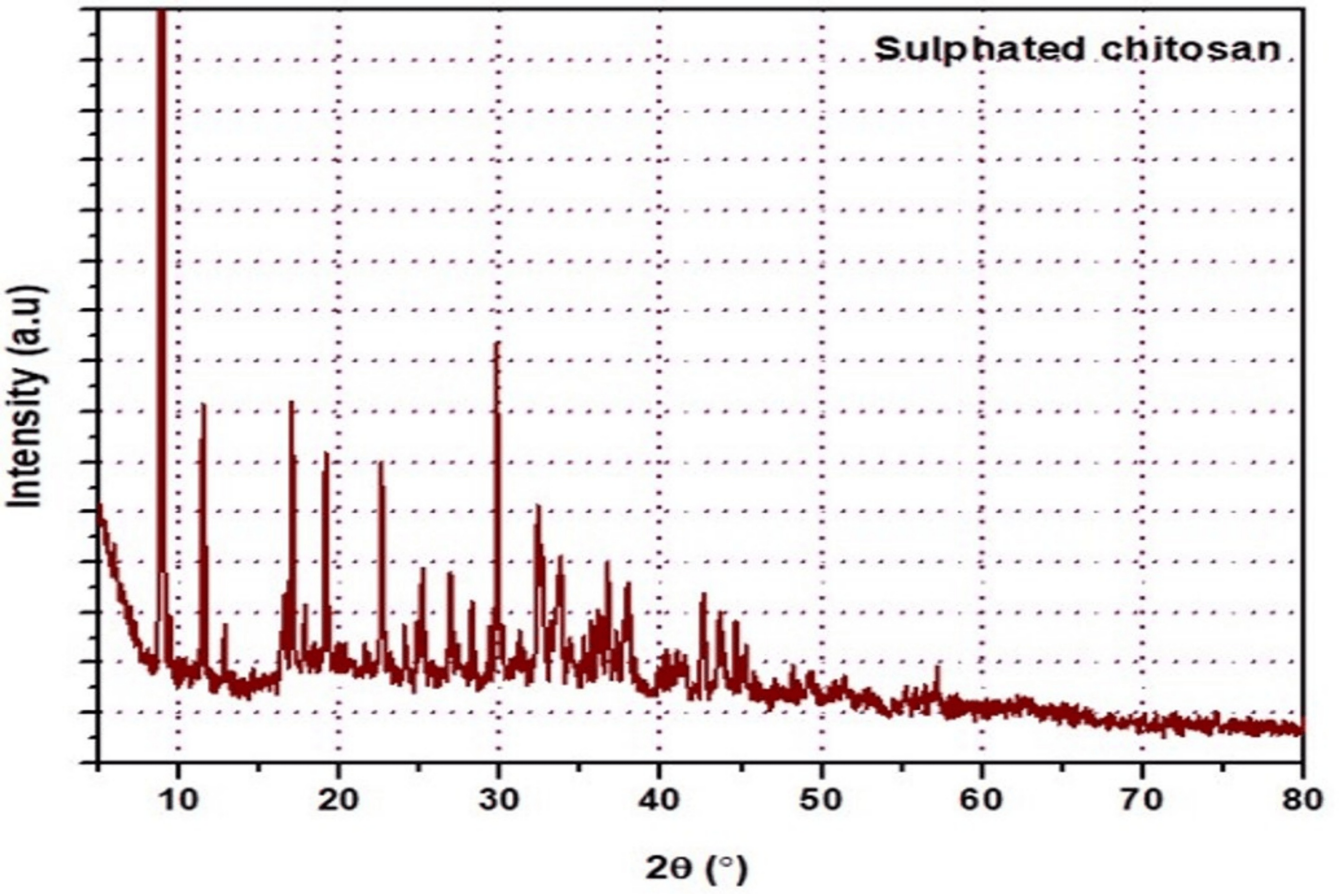
X-ray diffraction of graph of sulfated chitosan from internal bone of Sepiella inermis

XRD investigations of the sulfated chitosan showed extremely wide peaks at 2θ=10° (Figure 3). Weak peaks were observed in chitosan sulfate at 2θ values of 20° and 30°. Nevertheless, the relatively broad peak at 2θ=20° in sulfated chitosan got weaker, and the peak observed at 2θ=10° vanished.

*In vitro* anticoagulant assays

**Figure 4:**
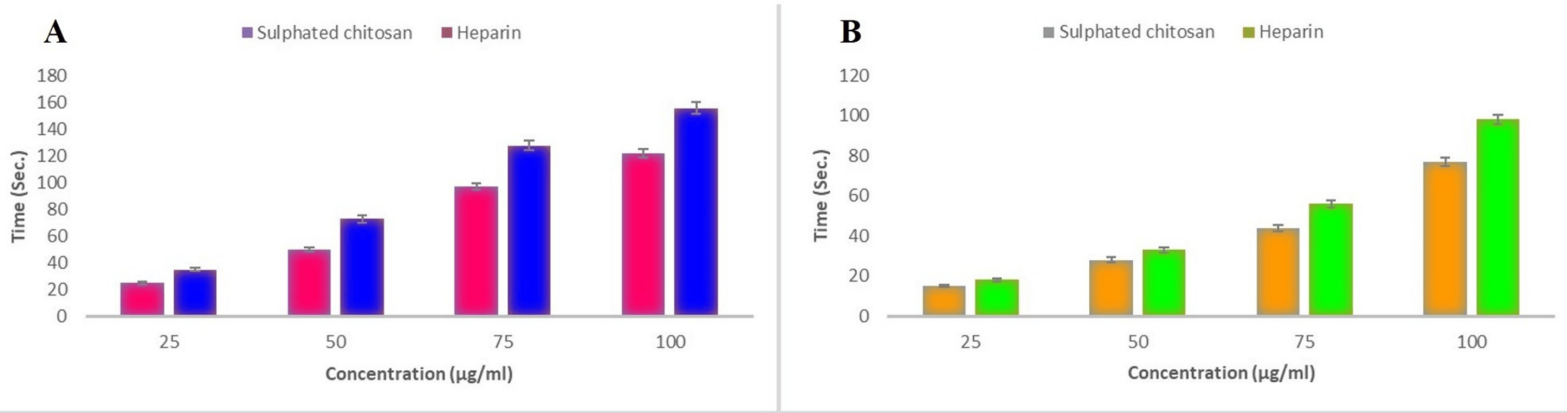
In vitro anticoagulant activity of sulphated chitosan from the internal bone of S. inermis (A: APTT, B: PT) APTT: activated partial thromboplastin time; PT: prothrombin time

Figure 4 suggests that sulfated chitosan and heparin, the commercial anticoagulants used in practice and procedures, have comparable anticoagulant activities. The results indicate that the sulfated chitosan exhibited APTT and PT values of 6.98 IU/mg and 1.77 IU/mg, respectively, whereas the standard showed values of 7.27 IU/mg and 1.98 IU/mg for APTT and PT, respectively. Sulfated chitosan exhibited statistically significant (P <0.05) different levels of anticoagulant properties between the four different concentrations tested.

## Discussion

The skeletal structure of chitin is strikingly similar to that of heparin; thus, researchers have focused on using chitin for biological purposes [16]. To varying degrees, deacetylation of chitosan is a crucial step that leads to the production of derivatives that resemble heparin. The sulfated chitosan of *Doryteuthis singhalensis* pen showed a transmittance peak between 3428 cm^-1^ and 470 cm^-1^ in the FT-IR spectrum. The absorption bands in the spectrum correspond to extracted sulfated chitosan [17,18]. The 3395 and 2923 cm^−1^ absorption bands indicated the presence of hydroxyl groups and C-H bonds in the sugar ring [19]. The acidic cycles were confirmed by the signals at 1027 cm^-1^ and 1144 cm^−1^, which matched the C-O-C, C-OH, and C-C vibrations, respectively. Another study found that the peaks at 1610 cm-1 in sulfated chitosan from *Ulva pertusa* were caused by the stretching motion of CHO and C=O bonds [20,21]. The spectrum of sulfur-treated chitosan showed peaks at 669.30 cm^-1^ and 1161.64 cm^−1^, which indicated the presence of sulfo groups. These peaks were related to the bonding of sulfur with other elements. The N-S=O bond stretched unevenly, as indicated by the strong absorption at 1161 cm^−1^ in the FT-IR spectrum. This resembled the structure of sulfated chitosan in the FT-IR spectral study. It exhibits a peak at 1160 cm^−1^ [22]. Because of the sulfo group, sulfated chitosan from the shell of *Donax scortum*, a type of shellfish, was found to have absorption peaks at 668.90 cm^−1^ and 1134.36 cm^−1^ [5]. The detected peaks (993.12 cm^-1^, 1160.19 cm^-1^, 1376.79 cm^-1^, and 1651.13 cm^-1^) in the current investigation are also included.

FESEM was used to examine the morphology of sulfated chitosan, and the results are displayed in Figure 2, along with photographs of the chitosan at two different magnifications and at various locations. The extracted sulfated chitosan had a structure resembling fibrils. Compared with our study, fibril structures in specific sulfated chitosan components were immediately identifiable. As the magnification of the sulfated chitosan increased, disintegrating flakes with fibril structures were also observed in the chitosan of crabs [23] and cuttlefish, *Sepia kobiensis* [24].

When we compared our X-ray diffraction (XRD) results with those of a previous study [25], significant differences were observed. In a previous study, chitosan showed peaks at 2θ = 9.8° and 20.8°, whereas cuttlefish chitosan showed clear crystalline peaks at 2θ = 20.7°. In contrast, two weak peaks at approximately 2θ = 20° and 30° were complemented by large peaks at 2θ = 10° and 2θ = 20° in the XRD analysis of sulfated chitosan. These differences imply that the sulfated chitosan in our investigation has special structural properties, most likely related to good compatibility, which results in the creation of a porous xerogel network.

Both non-fractionated heparin and derivatives of sulfated chitosan accelerate thrombin inactivation by creating an equimolar complex with antithrombin III [26, 27]. The sulfate group present in sulfated chitosan may be the cause of its anticoagulant properties. Furthermore, the anticoagulant characteristics of sulfated chitosan were significantly influenced by the location of the sulfate group. Additionally, it was shown that their anticoagulant actions work by the same mechanism as heparin, which directly and indirectly inhibits thrombin and antithrombin III activity, which in turn reduces Factor Xa activity [19, 28]. Similar to *S. inermis*, some mollusks, including *Sepioteuthis lessoniana*, have been shown to exhibit anticoagulant properties [10]. For the purpose of medication distribution, chitosan derived from *Sepia kobiensis *cuttlebones is another possible source of anticoagulant action [21]. Similarly, *Sepia pharaonis *had lower APTT and PT activities with sulfate chitosan (as 6.96 IU/mg of APTT and 1.93 IU/mg of PT) than our present findings [17]. Similar to this, another researcher demonstrated the anticoagulant activity of the sulfated chitosan extracted from the pen of squid *Doryteuthis singhalensis*, revealing values of 1.85 IU/mg (PT) and 6.91 IU/mg (APTT) [29]. Although the sulfated chitosan produced in this study under semi-heterogeneous conditions from the cuttlebone of *S. inermis* had a greater anticoagulant potential at 6.98 IU/mg of APTT and 1.77 IU/mg of PT. The goal of this study was to determine how structurally modified sulfated chitosan affects its anticoagulant action. The results also corroborate the idea that the sulfate group location of sulfated chitosan enhances the effectiveness of APTT and PT.

Limitation 

It is possible that the results cannot be applied to other chitosan sources because this study relied on only one species, *S. inermis.* The results may also be difficult to reproduce due to process variations in extraction and sulfation, which can alter the yield and efficiency. Further *in vivo* investigations are needed to confirm the efficiency and safety of anticoagulant assays, as their in vitro nature may not completely duplicate the complicated physiological conditions *in vivo*. Crucial considerations for the therapeutic use of sulfated chitosan, such as its immunogenicity and toxicity, were not addressed in this study. The last point that was left out was the economic feasibility and scalability of producing sulfated chitosan from the internal bone of *S. inermis*.

## Conclusions

In this study, we examined the anticoagulant properties of sulfate chitosan, synthesized from *S. inermis*. Heparin has been used as an anticoagulant since several years. Heparin is known to have several unfavorable side effects, including thrombocytopenia and an increased risk of bleeding. Therefore, it is necessary to find alternatives to the extensively used anticoagulant medications. Sulfated chitosan exhibits significant potential for applications in drug delivery systems, medical devices, and therapeutic anticoagulants. It is also a desirable option for the development of biomaterials with integrated anticoagulant characteristics, owing to its natural origin and biocompatibility. Sulfated chitosan showed similar anticoagulant properties *in vitro*, indicating its promise as a natural and inexpensive substitute for heparin, which remains the standard treatment.
